# Interlayer Architecture Modulates Water State and Transport in Chitosan Aerogels for Solar-Driven Interfacial Evaporation

**DOI:** 10.3390/nano16211413

**Published:** 2026-11-03

**Authors:** Fengxia An, Zihan Chen, Binbin Wang, Ke Zhuang, Jiayun He, Shunde Zou, Xiaohuan Meng, Enmin Zong, Haopeng Luo, Huan Chen, Fang Jiang

**Affiliations:** 1 State Key Laboratory of Low-Carbon Smart Coal-Fired Power Generation and Ultra-Clean Emission, China Energy Science and Technology Research Institute Co., Ltd., Nanjing 210023, China; 2 Key Laboratory of Energy Thermal Conversion and Control of Ministry of Education, School of Energy and Environment, Southeast University, Nanjing 210096, China; 3 Key Laboratory of Jiangsu Province for Chemical Pollution Control and Resources Reuse, School of Environmental and Biological Engineering, Nanjing University of Science and Technology, Nanjing 210094, China; 4 Zhejiang Key Laboratory for Restoration of Damaged Coastal Ecosystems, School of Life Sciences, Taizhou University, Taizhou 318000, China

**Keywords:** solar-driven interfacial evaporation, chitosan-based photothermal aerogel, lamellar architecture, water-state regulation, apparent evaporation enthalpy

## Abstract

Solar-driven interfacial evaporation offers a sustainable route to freshwater production, yet the role of evaporator microstructure in regulating water state and transport remains insufficiently understood. Here, chitosan-based photothermal aerogels with tunable lamellar architectures were fabricated by freeze-drying, using carbon nanotubes as broadband solar absorbers. Varying the chitosan concentration modulated the interlayer spacing and associated water state within the aerogels. The optimized CS2.5 aerogel exhibited the highest intermediate-water proportion and the lowest apparent evaporation enthalpy of 1652.1 kJ kg^−1^, while maintaining solar absorptance above 97.8%. Under 1 sun irradiation, CS2.5 achieved an evaporation rate of 2.33 kg m^−2^ h^−1^ and maintained stable performance in NaCl solutions of up to 15 wt%, organic-contaminated water, and prolonged cycling tests. Idealized COMSOL Multiphysics 6.3 simulations indicated that a moderate interlayer spacing promoted a uniform velocity distribution and smooth pressure gradient, thereby facilitating water transport. During six consecutive days of outdoor operation, the evaporator delivered daily evaporation yields of 1.38–2.30 kg m^−2^ under fluctuating environmental conditions. These findings provide insights into coordinating lamellar architecture, water-state regulation, and fluid transport for efficient solar evaporation.

## 1. Introduction

Freshwater scarcity has become an increasingly pressing global challenge owing to population growth, industrial expansion, and the uneven spatial distribution of water resources [1,2,3,4,5]. Conventional desalination technologies, including thermal distillation and membrane-based separation, can provide reliable freshwater supplies but are often constrained by high energy consumption, operational costs, membrane fouling, or concentrated brine generation. Solar-driven interfacial evaporation has emerged as a promising alternative because it directly converts abundant solar energy into localized heat for water evaporation and freshwater recovery [4,5,6]. Unlike conventional bulk-water heating, this approach confines thermal energy to the air–water interface, thereby suppressing heat dissipation to the underlying water and improving solar-energy utilization [7]. Moreover, the phase-change separation process can effectively retain dissolved salts, heavy metals, and many non-volatile organic contaminants, demonstrating considerable potential for decentralized desalination and water purification [8].

Photothermal aerogels have attracted increasing attention because their low density, high porosity, tunable surface chemistry, and interconnected pore networks enable their use in atmospheric water harvesting, solar–thermal energy storage, oil–water separation, and solar steam generation [9,10,11,12]. For solar-driven interfacial evaporation, an effective aerogel evaporator must simultaneously achieve broadband light absorption, localized thermal confinement, continuous water replenishment, and rapid vapor release [13,14]. These processes are strongly influenced by the internal pore architecture. Accordingly, structurally graded channels, interconnected pores, layered configurations, and gradient porous networks have been developed to regulate the coupled transport of water and heat [15,16,17,18,19]. Vertically aligned channels, for example, can provide direct pathways for capillary water transport and reduce the distance required for water replenishment [20], whereas ice-templating and ice-seed-tailoring strategies enable the construction of anisotropic or hierarchically organized pore networks [21]. Nevertheless, rapid water transport does not necessarily guarantee optimal evaporation performance. Insufficient water replenishment can cause local dehydration and interrupt evaporation, whereas excessive water transport may increase conductive heat loss and decrease the interfacial temperature. Therefore, balancing water supply and thermal localization is essential for sustained and energy-efficient evaporation.

Chitosan is a naturally derived polysaccharide with abundant hydroxyl and amino groups, favorable hydrophilicity, good film-forming capability, and versatile chemical functionality [22,23,24]. These characteristics make chitosan a suitable framework for constructing porous photothermal materials. More importantly, its hydrophilic functional groups can interact with water molecules through hydrogen bonding, potentially altering the organization, mobility, and evaporation behavior of water confined within the polymer network. Several chitosan-based evaporators have demonstrated promising solar evaporation performance. Liu et al. [25] developed an integrated Janus chitosan aerogel comprising a carbonized hydrophobic photothermal layer and a hydrophilic insulating layer, achieving an evaporation rate of 1.76 kg m^−2^ h^−1^ under 1 sun irradiation. Xue et al. [26] constructed a semi-encapsulated chitosan/carbon-nanotube aerogel in which the hydrophobic inner region enhanced floating stability and suppressed heat loss, resulting in an evaporation rate of 1.547 kg m^−2^ h^−1^. More recently, Qu et al. [27] fabricated a palygorskite–carbon-nanotube/chitosan aerogel with vertically aligned channels, enabling continuous water replenishment and stable evaporation under high-salinity conditions. These studies establish chitosan as a versatile platform for solar evaporation; however, they have mainly focused on compositional design, surface properties, macroscopic pore orientation, and thermal management.

Recent studies have further demonstrated that pore architecture plays a critical role in regulating light scattering, capillary water transport, heat transfer, and overall evaporation performance [28,29]. Zong et al. [30] constructed a porous cellulose aerogel through a non-freeze-drying strategy, in which the tailored pore structure enhanced internal light scattering and reduced heat dissipation. Guo et al. [31] developed an alginate-fiber aerogel with vertically aligned channels and hierarchical pores; the vertical channels facilitated continuous water transport, while the hierarchical porous network improved light absorption through multiple scattering. Nevertheless, total porosity or average pore size alone cannot fully describe the transport characteristics of layered aerogels. Materials with similar overall porosities may exhibit markedly different interlayer spacings, pore connectivity, water–polymer interactions, and flow resistance. In particular, the relationship among interlayer spacing, confined-water state, apparent evaporation enthalpy, and water-transport behavior remains insufficiently understood. Excessively large interlayer channels may weaken water confinement and increase heat transfer to the bulk water, whereas overly narrow channels may increase flow resistance and strengthen the binding of water molecules to hydrophilic pore walls. Clarifying this structure–water-transport relationship is therefore important for moving from empirical pore optimization toward the rational design of porous photothermal evaporators.

Herein, chitosan-based photothermal aerogels with tunable lamellar architectures were fabricated through freeze-drying, using carbon nanotubes as broadband solar absorbers. The chitosan concentration was varied to modulate the interlayer architecture and the associated water state and transport behavior. As illustrated in Figure S1, the proposed evaporation system integrates solar absorption, capillary water supply through interconnected lamellar channels, localized interfacial evaporation, and subsequent condensation for freshwater collection. Dissolved salts remain in the feed-side liquid, while the condensate is collected separately. Local salt enrichment at the evaporation interface may promote ion back-diffusion toward the bulk reservoir through the connected water-filled channels. In this batch configuration, residual concentrate remains in the feed reservoir and requires separate collection and appropriate treatment after operation; extended operation would require controlled concentrate withdrawal and feed replenishment. Its potential applications include decentralized desalination and the purification of water containing nonvolatile organic contaminants. With further development of the integrated evaporation–condensation system, this approach could offer a solar-powered alternative to small-scale electrically or fuel-heated distillation units. The resulting aerogels were systematically investigated in terms of their morphology, pore characteristics, optical absorption, wettability, photothermal response, intermediate-water proportion, apparent evaporation enthalpy, and solar evaporation performance. Idealized numerical simulations were further employed to examine the effects of interlayer spacing on the internal velocity and pressure distributions. Finally, the environmental adaptability and practical potential of the optimized evaporator were evaluated in saline water, organic-contaminated water, prolonged cycling tests, and outdoor evaporation experiments. This study highlights the coupled roles of lamellar architecture, water-state regulation, and fluid transport, providing insights into the structural design of efficient and environmentally adaptable solar evaporators.

## 2. Experimental Sections

### 2.1. Material Fabrication

The chitosan-based photothermal aerogels were prepared by freeze-drying using 300, 400, 500, 600, or 700 mg of chitosan. Briefly, 200 μL of acetic acid was added to 20 mL of deionized water, followed by the addition of 10 mg of CNTs and the specified amount of chitosan. The mixture was stirred at 50 °C for 5 h to obtain a homogeneous precursor dispersion. Subsequently, the dispersion was transferred into a cylindrical tube with an internal diameter of 3.5 cm and a height of 5 cm and frozen overnight. After complete solidification, the samples were freeze-dried at −40 °C for 48 h and subsequently treated with 0.5 mol L^−1^ NaOH solution for 3 h to obtain the final chitosan-based photothermal aerogels. The samples were designated CS_x_, where x represents the nominal chitosan concentration in % (*w*/*v*), calculated relative to the initially added 20 mL of deionized water. Accordingly, chitosan masses of 300, 400, 500, 600, and 700 mg correspond to CS_1.5_, CS_2.0_, CS_2.5_, CS_3.0_, and CS_3.5_, respectively.

### 2.2. Characterization

Scanning electron microscopy (SEM, Quanta 400 FEG, FEI Company, Hillsboro, OR, USA) measurement was conducted using MonoCL^3+^ combined with a cathode fluorescence spectrometer, with the wavelength range being 160–930 nm. Ultraviolet–visible diffuse reflectance spectra (UV-Vis DRS) in the range of 250–2500 nm were recorded on an ultraviolet–visible spectrophotometer (UV3600, Shimadzu Corporation, Kyoto, Japan). Raman spectra were acquired using a LabRAM HR Evolution spectrometer (HORIBA Jobin Yvon, Palaiseau, France) with a 532 nm excitation laser. In situ shell-isolated nanoparticle-enhanced Raman spectroscopy (SHINERS) measurements were performed under controlled temperature and atmosphere.

The surface hydrophilicity of the photothermal aerogel was characterized by a contact angle goniometer (OCA20, DataPhysics Instruments GmbH, Filderstadt, Germany). The surface temperature distribution of the samples was visually analyzed by capturing thermal images using an infrared thermal imager (FLIR ONE Pro, FLIR Systems, Inc., Wilsonville, OR, USA). The heat absorbed by the aerogel during the heating process was measured using a differential scanning calorimeter (DSC25, TA Instruments, New Castle, DE, USA), enabling the precise quantification of the energy required for the liquid-to-vapor phase transition of water.

The mercury pressure test was conducted using the AutoPore IV 9600 automatic mercury porosimeter (Micromeritics Instrument Corporation, Norcross, GA, USA). The instrument featured a maximum operating pressure of 413 MPa and a pore size measurement range from 3 nm to 370 μm. The calculation formula is as follows:

(1) $$P\;=\;-\frac{2\sigma \;\cos\alpha }{r}$$

where P is the pressure required to compress mercury into a pore with a radius of ‘r’, and the unit is Pa. r represents the pore radius, with the unit being nanometers. σ represents the surface tension of mercury, with the unit being N m^−1^. α represents the wetting angle of mercury on the material, with the unit being degrees.

### 2.3. Solar-Driven Interfacial Evaporation Device

To evaluate the solar evaporation performance of the prepared materials, a solar-driven interfacial evaporation device based on the photothermal aerogel was designed and fabricated. The photothermal aerogel was immobilized on a polystyrene foam substrate, which was then made to float on the water surface. The capillary effect of the aerogel enabled continuous upward transport of bulk water from the bottom to the evaporation surface. Carbon nanotubes were uniformly dispersed within the aerogel matrix to serve as the light-absorbing and photothermal conversion component. With light irradiation from the top, the upper surface of the aerogel acted as the primary evaporation interface. Moreover, lateral convection and heat conduction of the aerogel further enhanced the evaporation process and realized efficient utilization of the heat generated by photothermal conversion.

## 3. Results and Discussion

### 3.1. Preparation and Characterization of Cs_x_

**Figure 1:**
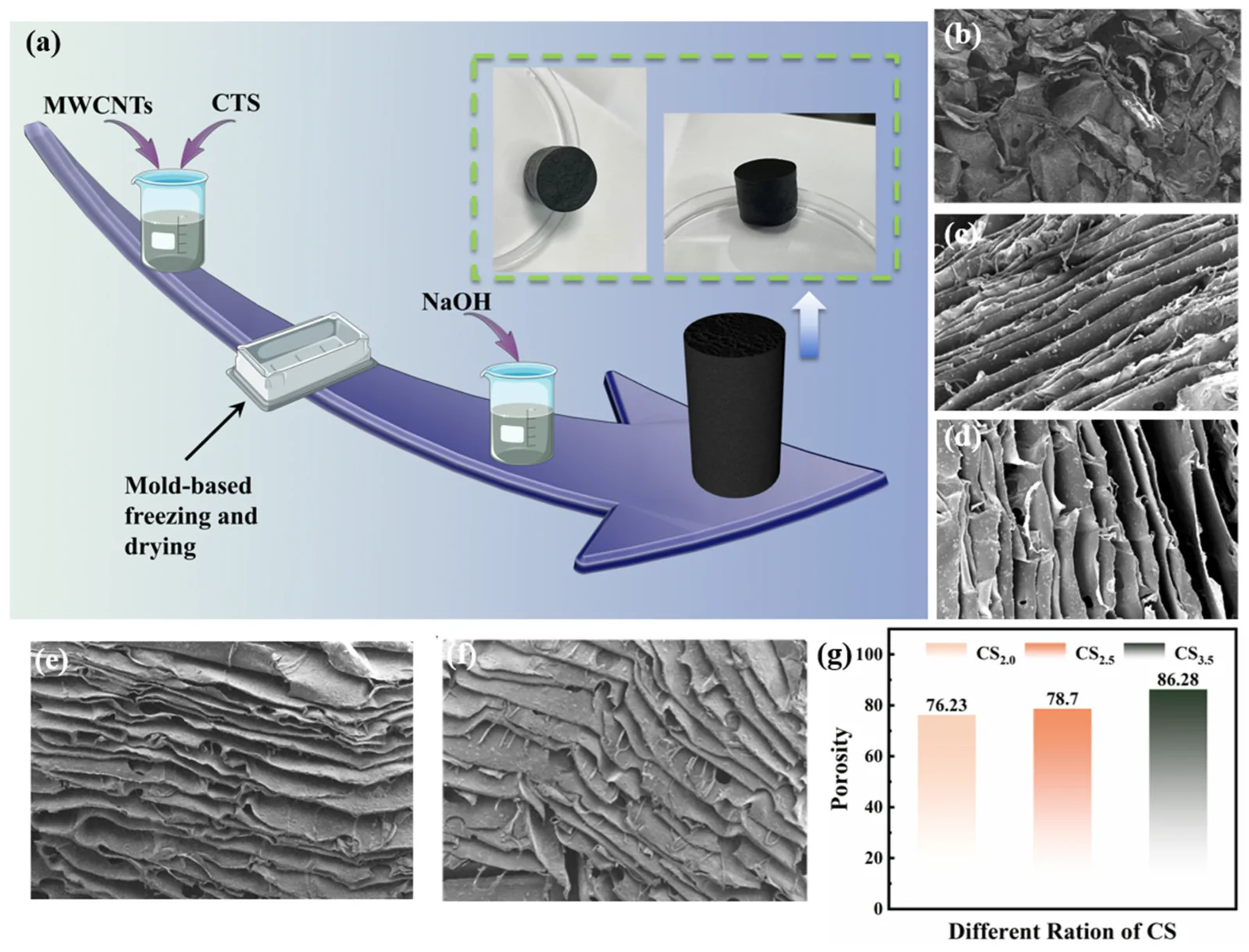
(**a**) The preparation process of CS_x_. SEM images of CS_x_ evaporators: (**b**) CS_1.5_, (**c**) CS_2.0_, (**d**) CS_2.5_, (**e**) CS_3.0_, and (**f**) CS_3.5_. (**g**) Porosity of the CS_x_ evaporator.

To investigate the effect of chitosan amount in aerogels on the interfacial evaporation performance of aerogel-based evaporators, we fabricated integrated photothermal evaporators by loading multi-walled carbon nanotubes (MWCNTs) onto chitosan aerogels. The CS_x_ was prepared by freeze-drying (Figure 1a). The microstructures of chitosan aerogels with different concentrations were characterized by scanning electron microscopy (SEM). As shown in the SEM images of the chitosan aerogels (Figure 1b–f), all aerogels except CS_1.5_ formed a layered structure composed of stacked thin sheets, which is a typical morphology formed during freeze-drying due to ice sublimation. The interlayer spacings of the CS_2.0_, CS_2.5_, CS_3.0_, and CS_3.5_ aerogels were 100 µm, 79 µm, 60 µm, and 30 µm, respectively, indicating that the interlayer spacing decreased and the structure became denser with the increase in chitosan concentration. The high-magnification SEM images of the CS_2.5_ aerogel are presented in Figure S2. As shown in Figure S2a,b, elongated fiber-like structures and short rod-like or flake-like particle clusters can be observed on the chitosan framework. The fiber-like structures exhibit morphological features consistent with CNT-containing composites, while the presence of local particle clusters suggests a heterogeneous microstructure. These observations provide additional insight into the morphology of the CNT/chitosan composite aerogel. The CNTs serve as photothermal components, contributing to the broadband solar absorption of the prepared evaporator.

To further investigate the effect of chitosan amount on the pore structure of the aerogels, mercury intrusion porosimetry was used to determine their porosity. As shown in Figure 1g, the porosities of the CS_2.0_, CS_2.5_, and CS_3.5_ aerogels were 76.23%, 78.70%, and 86.28%, respectively, which were consistent with the SEM characterization results. These porosity values are comparable to those reported for chitosan-based aerogels fabricated via freeze-drying in the literature, which typically range from 70% to over 99% depending on the specific formulation and processing conditions [32,33,34]. Notably, although CS_3.5_ exhibited the highest porosity among the samples, CS_2.5_ achieved the highest evaporation rate in subsequent tests. This observation suggests that porosity alone is not the dominant factor determining evaporation performance; rather, the water state and interlayer spacing play a more critical role, as will be discussed in the following sections.

The FTIR spectrum of CS_2.5_ (Figure S3) exhibits a broad absorption band at approximately 3200–3500 cm^−1^, corresponding to overlapping O-H and N-H stretching vibrations, together with aliphatic C-H stretching bands in the 2850–2950 cm^−1^ region. The band near 1650 cm^−1^ is associated with the amide I vibration of residual N-acetyl groups and may include a contribution from adsorbed water, whereas the neighboring band near 1590 cm^−1^ is attributed to NH_2_ bending. The absorption near 1150 cm^−1^ and the strong bands within 1000–1100 cm^−1^ correspond to C-O-C stretching and C-O/polysaccharide skeletal vibrations, respectively. These spectral features identify the hydroxyl- and amino-containing chitosan framework, whose polar groups provide hydrogen-bonding sites for water and contribute to the wettability of the lamellar channels.

**Figure 2:**
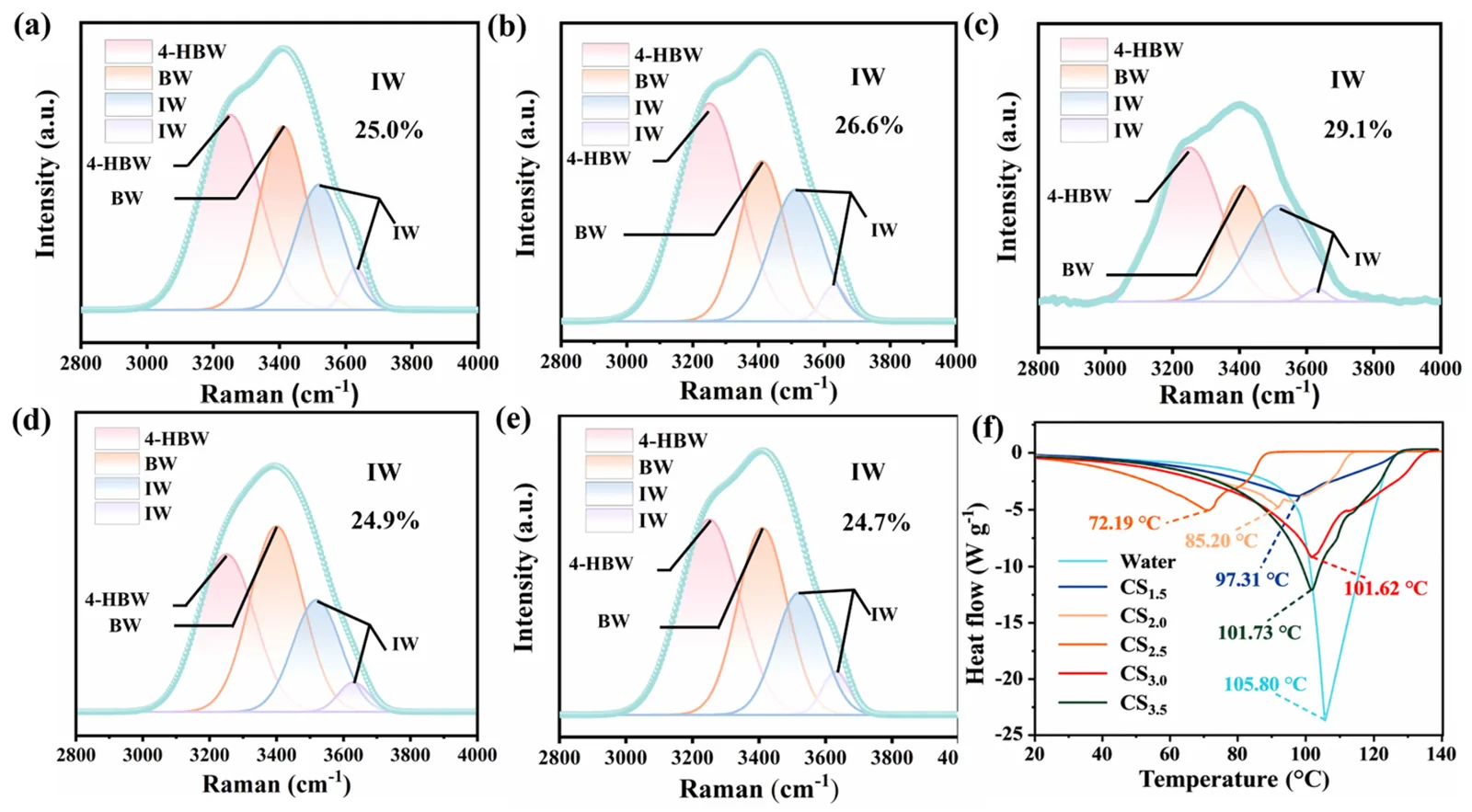
Raman fitting curves depicting the state of water molecules in the CS_x_ evaporators: (**a**) CS_1.5_, (**b**) CS_2.0_, (**c**) CS_2.5_, (**d**) CS_3.0_, and (**e**) CS_3.5_. DSC test results and the difference in evaporation enthalpy of the CS_x_ evaporators: (**f**) DSC test results.

The hydrogen bonding interaction between the polymer molecular network and water molecules has a significant impact on the evaporation enthalpy. During natural evaporation, water molecules need to overcome the hydrogen bonding forces between themselves. Free water (4-HBW) refers to water molecules forming a tetrahedral hydrogen-bonded structure with four other water molecules. The strong hydrogen bonding between the hydrophilic groups of the chitosan polymer and water molecules weakens the hydrogen bonding among water molecules, thus forming intermediate water (IW). A higher proportion of intermediate water corresponds to lower energy required for evaporation. As shown in Figure 2a–e, the Raman spectral range of 3000 cm^−1^ to 4000 cm^−1^ corresponds to the hydrogen bonding vibration of water molecules, where the peak at 3250 cm^−1^ represents 4-HBW, the peak at 3410 cm^−1^ corresponds to bound water (BW), and the peaks at 3520 cm^−1^ and 3630 cm^−1^ are attributed to IW. The proportion of intermediate water in the aerogels was calculated by the ratio of peak area, which was 25.0%, 26.6%, 29.1%, 24.9%, and 24.7% for the series of samples, respectively. Among the CS_x_ samples, CS_2.5_ exhibited the highest proportion of intermediate water, thus requiring the least energy to evaporate a unit mass of water and resulting in the fastest evaporation rate. This trend is consistent with recent reports on bio-based aerogels for solar interfacial evaporation, where the presence of abundant hydrophilic groups (hydroxyl, amine, and carboxyl) in the polymer network promotes the formation of intermediate water and effectively reduces the evaporation enthalpy. Wang et al. [35] demonstrated that chitosan/cellulose-based aerogels achieved a low evaporation enthalpy of 1103 J g^−1^ by increasing the intermediate water content through hydrogen bonding interactions, further confirming that the regulation of water state is a key strategy for enhancing evaporation performance.

Differential scanning calorimetry (DSC) was employed to measure the evaporation enthalpy of water in different aerogels (Figure 2f and Figure S4). The results showed that the aerogel matrix effectively reduced the evaporation enthalpy of water, and CS_2.5_ had the lowest evaporation enthalpy of 1652.1 kJ kg^−1^, which was consistent with the Raman spectral analysis results. The combined results of Raman and DSC characterizations clarified the mechanism by which the interlayer spacing affects the evaporation rate: when the interlayer channels are too wide, water molecules tend to form a complete hydrogen bonding network similar to free water, leading to higher energy required for evaporation; on the contrary, when the interlayer spacing is too small, water molecules have a strong interaction with the functional groups (e.g., -OH and -COOH) on the pore walls, which restricts their molecular motion and increases the binding energy, thus requiring more energy for evaporation.

**Figure 3:**
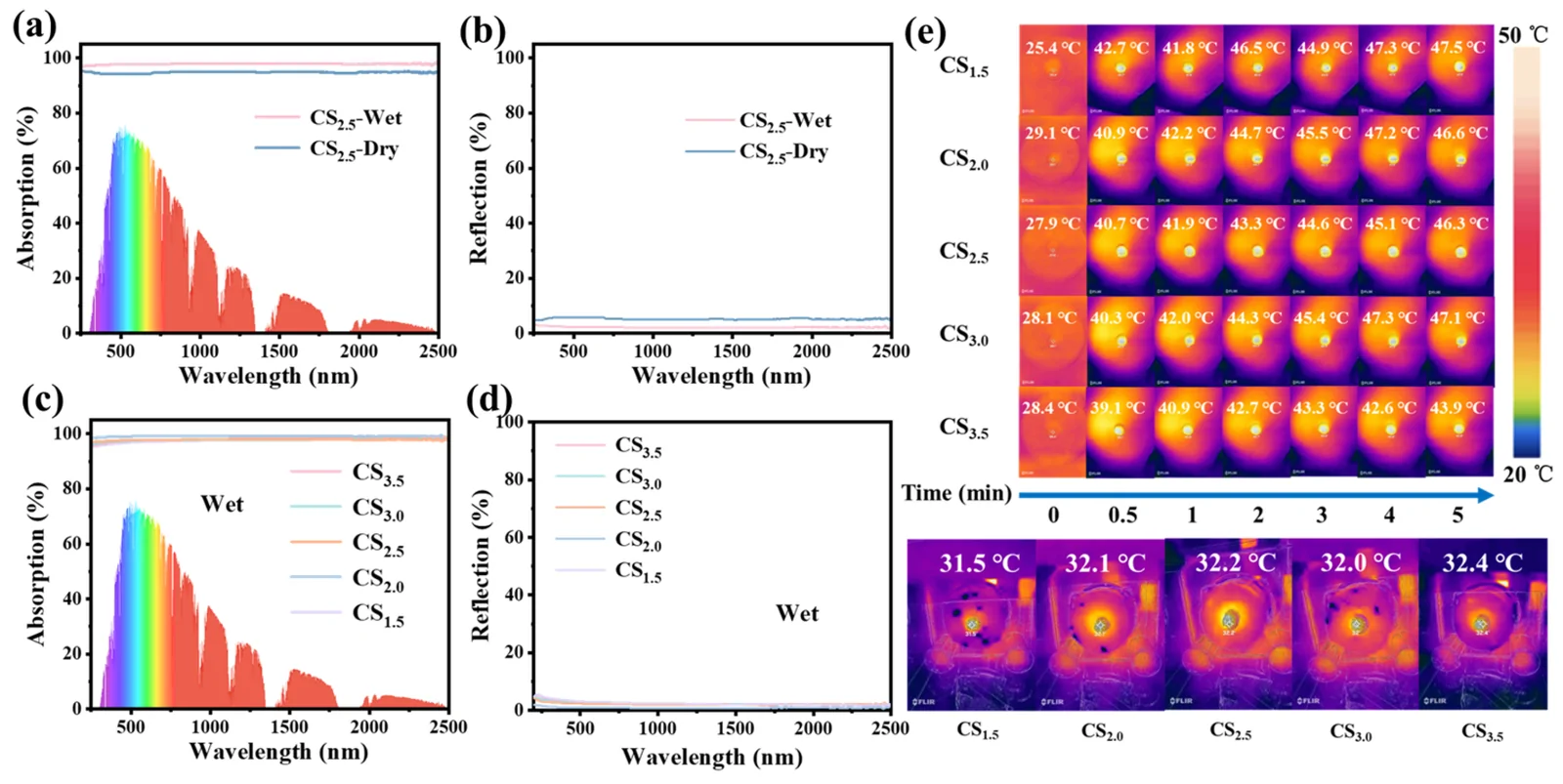
(**a**) Absorption and (**b**) reflection spectra of CS_2.5_ under wet and dry conditions. (**c**) Absorption and (**d**) reflection spectra of CS_x_ evaporators under wet conditions. (**e**) Infrared thermographic images of CS_x_ evaporators (CS_1.5_, CS_2.0_, CS_2.5_, CS_3.0_, and CS_3.5_).

To evaluate the photothermal conversion ability of different evaporators, the solar radiation absorption properties of CS_1.5_, CS_2.0_, CS_2.5_, CS_3.0_, and CS_3.5_ aerogels in the wet state were characterized over the full spectral range of 250–2500 nm (Figure 3a–d). The results showed that the CS_2.5_ evaporator had a high average light absorption rate of 97.81% in the wet state, which was higher than its absorption rate in the dry state (94.81%). The light absorption rates of the different evaporators in the wet state were 97.56% (CS_1.5_), 99.09% (CS_2.0_), 97.82% (CS_2.5_), 97.73% (CS_3.0_), and 97.87% (CS_3.5_), all of which were above 97.56%, indicating excellent light absorption performance. This result suggests that the wet state generally enhances the light absorption ability of the aerogels, which is conducive to photothermal conversion and the formation of local thermal fields, while the differences in light absorption rates among different samples are negligible.

To investigate whether the differences in evaporation performance were related to the thermal response behavior, thermocouples and infrared thermal imaging were used to monitor the surface temperature changes in the evaporators under light illumination. Under the same light intensity (1 sun), all aerogel evaporators exhibited excellent photothermal response performance (Figure 3e), rapidly heating up to 46 °C in the dry state and 32 °C in the wet state. These results indicated that all aerogels could provide stable and efficient thermal energy for water evaporation. Notably, the surface temperatures of different evaporators (CS_1.5_, CS_2.0_, CS_2.5_, CS_3.0_, and CS_3.5_) in Figure S5 showed no significant difference in both dry and wet states, which further confirmed that thermal response behavior and heat localization capability were not the primary factors leading to the differences in evaporation rate.

**Figure 4:**
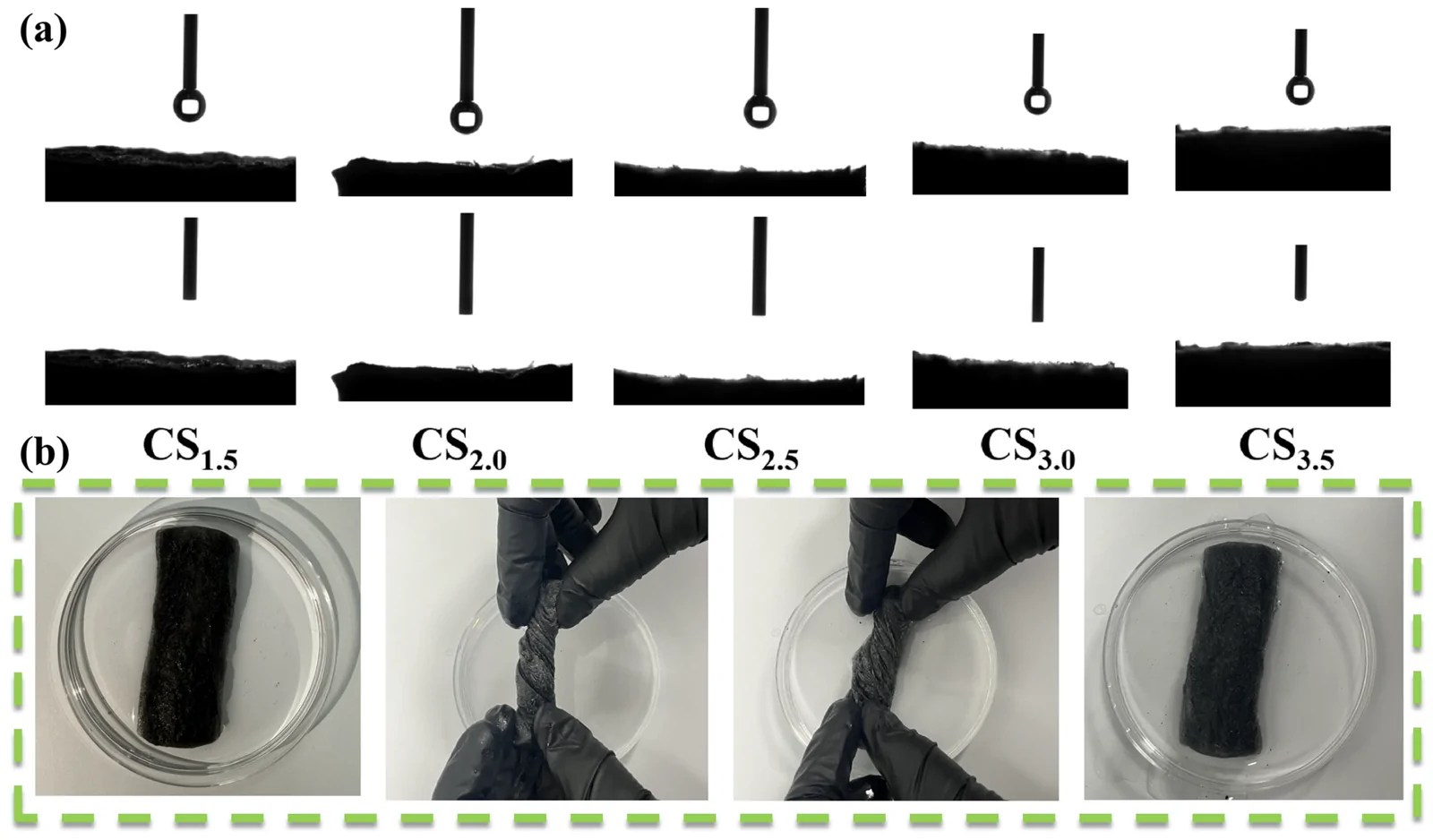
(**a**) Water contact angles for CS_x_ evaporators (CS_1.5_, CS_2.0_, CS_2.5_, CS_3.0_, and CS_3.5_). (**b**) Folding and retracting diagram of the CS_2.5_ evaporator.

The wettability of different evaporators was characterized by water contact angle measurements (Figure 4a). The results showed that water droplets rapidly penetrated the surfaces of all evaporators, indicating that all evaporators possessed excellent hydrophilicity. Meanwhile, no significant difference in wettability was observed among the samples, suggesting that wettability was not the dominant factor resulting in the differences in evaporation performance. In addition to wettability, the mechanical flexibility of the evaporators was also evaluated (Figure 4b). The CS_x_ evaporator exhibited good structural integrity and could be folded without cracking or delamination, and it quickly returned to its original shape upon release. This excellent flexibility facilitates practical handling and ensures stable performance under various deployment conditions.

After systematically characterizing how chitosan content affects the interlayer spacing, water state (intermediate water proportion), and evaporation enthalpy of chitosan aerogels, we concluded that CS_2.5_, possessing an optimal interlayer spacing and the highest proportion of intermediate water, achieved the lowest evaporation enthalpy and the highest evaporation rate. The similar performance in light absorption, photothermal response, and wettability among all CS_x_ samples further confirmed that the microstructure, especially the interlayer spacing and the corresponding water state, is the key factor leading to the differences in evaporation performance of the aerogel evaporators.

### 3.2. The Evaporation and Desalination Performance of Cs_x_

**Figure 5:**
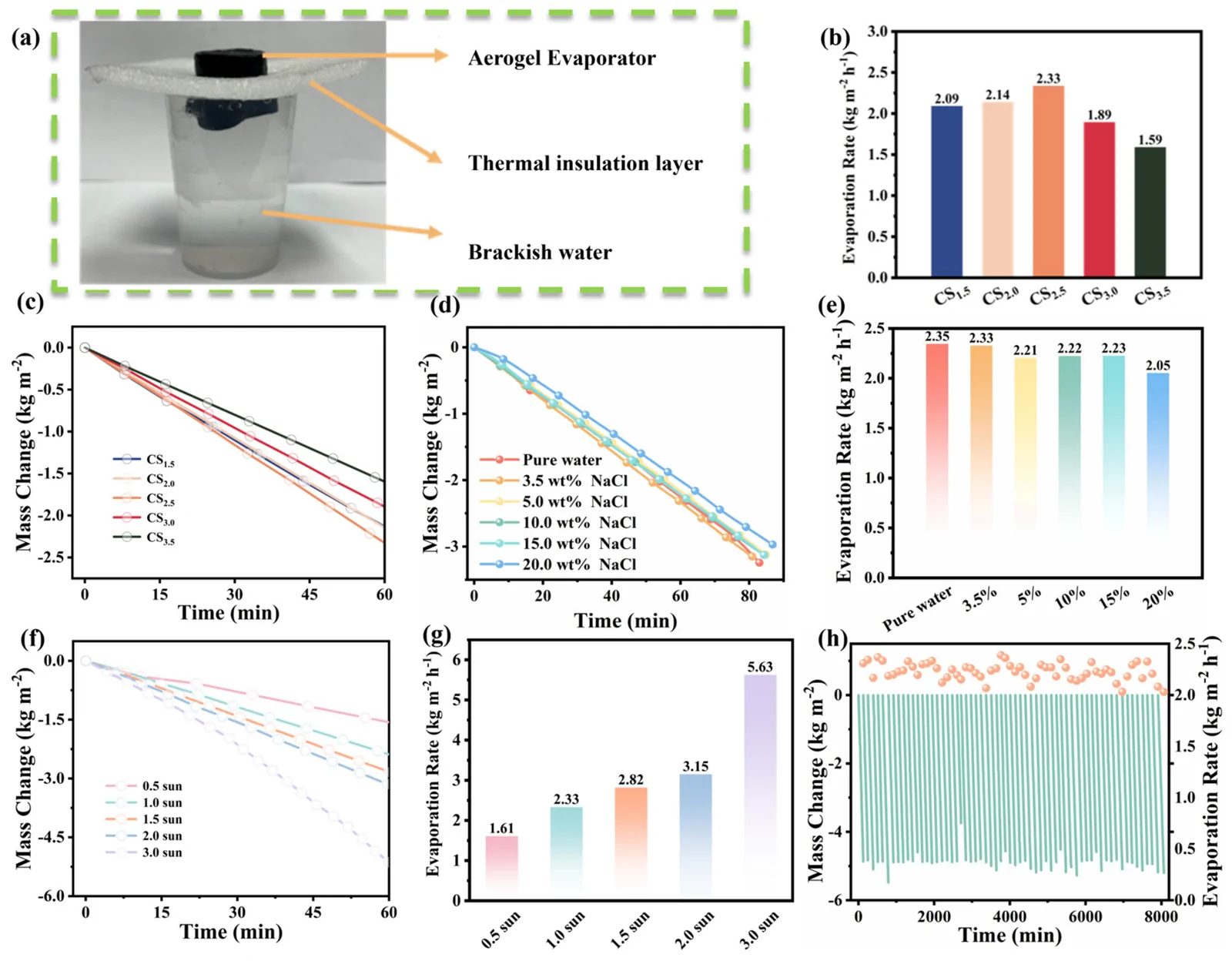
(**a**) Physical photo of chitosan aerogel photothermal evaporator. (**b**) Evaporation rates and (**c**) water mass change of CS_x_ evaporators (CS_1.5_, CS_2.0_, CS_2.5_, CS_3.0_, and CS_3.5_). (**d**) Water mass change and (**e**) evaporation rate of the CS_2.5_ evaporator at different NaCl concentrations. Evaporation performance of the CS_2.5_ evaporator characterized by (**f**) water mass loss and (**g**) evaporation rate under varying solar irradiances. (**h**) Water mass loss and evaporation rate curves of the CS_2.5_ evaporator over 60 operation cycles. The green curves represent water mass change (left y-axis), and the orange circles represent evaporation rate (right y-axis).

The evaporator structure of CS_x_ is shown in Figure 5a. Under one solar illumination, the temporal variation in water mass was recorded, and the evaporation rates of different evaporators were calculated accordingly. The evaporation rate exhibited a volcano-shaped dependence on the nominal chitosan concentration, reaching a maximum of 2.33 kg m^−2^ h^−1^ for CS_2.5_, prepared at a nominal chitosan concentration of 2.5% (*w*/*v*) (Figure 5b,c). No visible salt crystals developed on the exposed surface of CS_2.5_ during 72 h of continuous evaporation in 3.5 wt% NaCl solution (Figure S6). Cross-sectional SEM images acquired after direct freeze-drying without washing revealed crystalline deposits within the lamellar channels (Figure S7), consistent with the crystallization of saline liquid retained in the pores during sample preparation. During operation, the interconnected, water-filled channels support capillary replenishment and ion transport between the evaporation interface and the bulk reservoir. Evaporation-induced salt enrichment near the interface can establish a concentration gradient that promotes ion back-diffusion toward the less concentrated bulk solution. This redistribution may alleviate local salt accumulation and contribute to sustained evaporation performance without visible surface crystallization.

Salt is retained within the connected feed-side liquid, comprising the bulk reservoir solution and the pore liquid. Continued water removal progressively concentrates the residual feed, with supersaturation providing the driving force for salt nucleation and crystal growth. In the present batch configuration, the residual concentrate remains in the feed reservoir, while the condensate is collected separately (Figure S1). Concentrate collection after each batch, together with controlled withdrawal and feed replenishment during extended operation, is therefore necessary for salt management.

To evaluate the practical applicability and stability of the aerogel evaporator in complex water environments, the response of solar evaporators to different salt concentrations was investigated. In high-salinity water with NaCl concentrations ranging from 0 to 15 wt%, the evaporation rate of the CS_2.5_ evaporator remained stable at 2.21–2.35 kg m^−2^ h^−1^ without a significant decline (Figure 5d,e). Only when the NaCl concentration reached 20 wt% did the evaporation rate slightly decrease to 2.05 kg m^−2^ h^−1^, which was mainly due to the increased vapor pressure of the salt solution at high concentrations. The presence of NaCl enhanced the hydration interaction between water molecules and ions, thus hindering the escape of water molecules and the formation of water vapor. Meanwhile, the effects of different solar equivalents on evaporation performance were investigated. The evaporation performance of the CS_2.5_ evaporator was tested under radiation intensities ranging from 0.5 to 3 sun (Figure 5f,g). The results showed that the evaporation rate increased with the rise in radiation intensity, and the maximum evaporation rate of 5.63 kg m^−2^ h^−1^ was achieved under 3 sun irradiation. This result indicated that the CS_2.5_ evaporator possessed excellent thermal field regulation ability, which could maintain high energy utilization efficiency and stable evaporation performance under varying light intensity conditions.

The durability and reusability of the CS_2.5_ evaporator were evaluated over 60 consecutive photothermal evaporation cycles, corresponding to a total operating time of 8000 min (Figure 5h). The evaporation rate remained above 2.00 kg m^−2^ h^−1^ throughout the test without an appreciable decline, demonstrating its favorable long-term operational stability. As summarized in Table S1, the CS_2.5_ evaporator also exhibited a competitive evaporation rate of 2.33 kg m^−2^ h^−1^ under 1 sun irradiation compared with recently reported aerogel-based photothermal evaporators [35,36,37,38,39,40,41]. These results highlight the stable and competitive evaporation performance of CS_2.5_ and its potential for solar-driven desalination.

### 3.3. Simulation of Interlayer Spacing Regulating Water Transport

**Figure 6:**
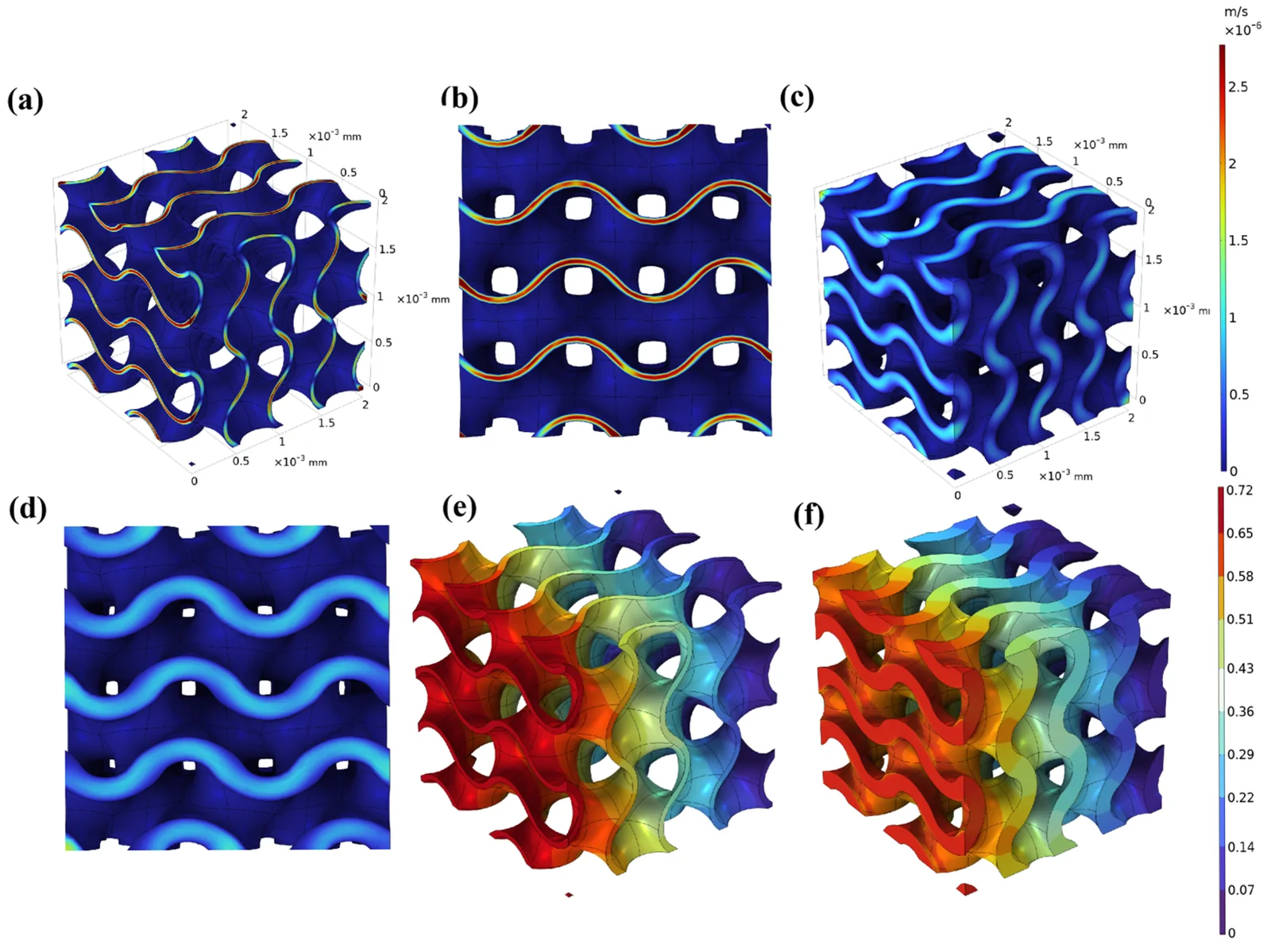
Three-dimensional overall views of water flow velocity distribution inside (**a**) CS_2.5_ and (**c**) CS_3.5_ chitosan aerogels; longitudinal section views of water flow velocity distribution inside (**b**) CS_2.5_ and (**d**) CS_3.5_ chitosan aerogels; 3D overall views of pressure distribution inside (**e**) CS_2.5_ and (**f**) CS_3.5_ chitosan aerogels.

To elucidate the regulatory mechanism of interlayer spacing on water transport behavior from the microscopic transport perspective, numerical simulations were performed using COMSOL Multiphysics to investigate the water flow velocity and pressure distribution of chitosan aerogels with different interlayer spacings. The detailed geometric parameters and boundary conditions applied in these simulations are provided in the Supplementary Information (Figures S8 and S9). To intuitively reveal the regulatory effect of interlayer spacing on water transport, quantitative analysis of the water flow velocity distribution in the 3D pores of two typical interlayer spacings (corresponding to CS_2.5_ and CS_3.5_ samples) was conducted. As shown in Figure 6a,c, the CS_2.5_ sample presents a uniform and continuous water flow velocity distribution without obvious sudden velocity changes or stagnant zones, indicating low water transport resistance and high transport efficiency. For the CS_3.5_ sample, the velocity distribution is highly uneven, with only a local high-velocity zone in the center of the pores, while the velocity near the pore walls drops sharply, forming a large-area, low-velocity stagnant zone and severely hindering water transport.

The longitudinal cross-sectional views (Figure 6b,d) further clarify the transport differences between the two structures. In the CS_2.5_ sample, the velocity distribution in the wavy pores is uniform with consistent vector directions, allowing water molecules to rise continuously and stably along the pores, achieving optimal capillary water transport efficiency. In the CS_3.5_ sample, the excessively compressed pores cause water flow extrusion. The velocity is high in the central region of the pores but drops sharply near the pore walls. This results in an extreme velocity gradient, with local velocity differences reaching an order of magnitude. Such a distribution disrupts the continuity of water transport and leads to insufficient capillary driving force.

The pressure field simulation further verifies the core mechanism of interlayer spacing regulating water transport and evaporation performance, as shown in Figure 6e,f. The CS_3.5_ aerogel (small interlayer spacing) exhibits an extremely uneven internal pressure gradient due to excessive pore compression, with a large-area, high-pressure zone (up to 0.73 Pa) at the inlet and a sharp pressure drop at the outlet, resulting in a local pressure difference exceeding 0.7 Pa. This creates extremely high water transport resistance and severely weakens the capillary driving force. In contrast, the CS_2.5_ aerogel (moderate interlayer spacing) shows a gentle and continuous internal pressure gradient, without local high-pressure or negative-pressure zones, with the overall pressure maintained in a reasonable range of 0.3–0.5 Pa. The moderate capillary driving force and minimum water transport resistance make it most conducive to the continuous and stable rise in water molecules along the pores.

### 3.4. Water Purification Performance

**Figure 7:**
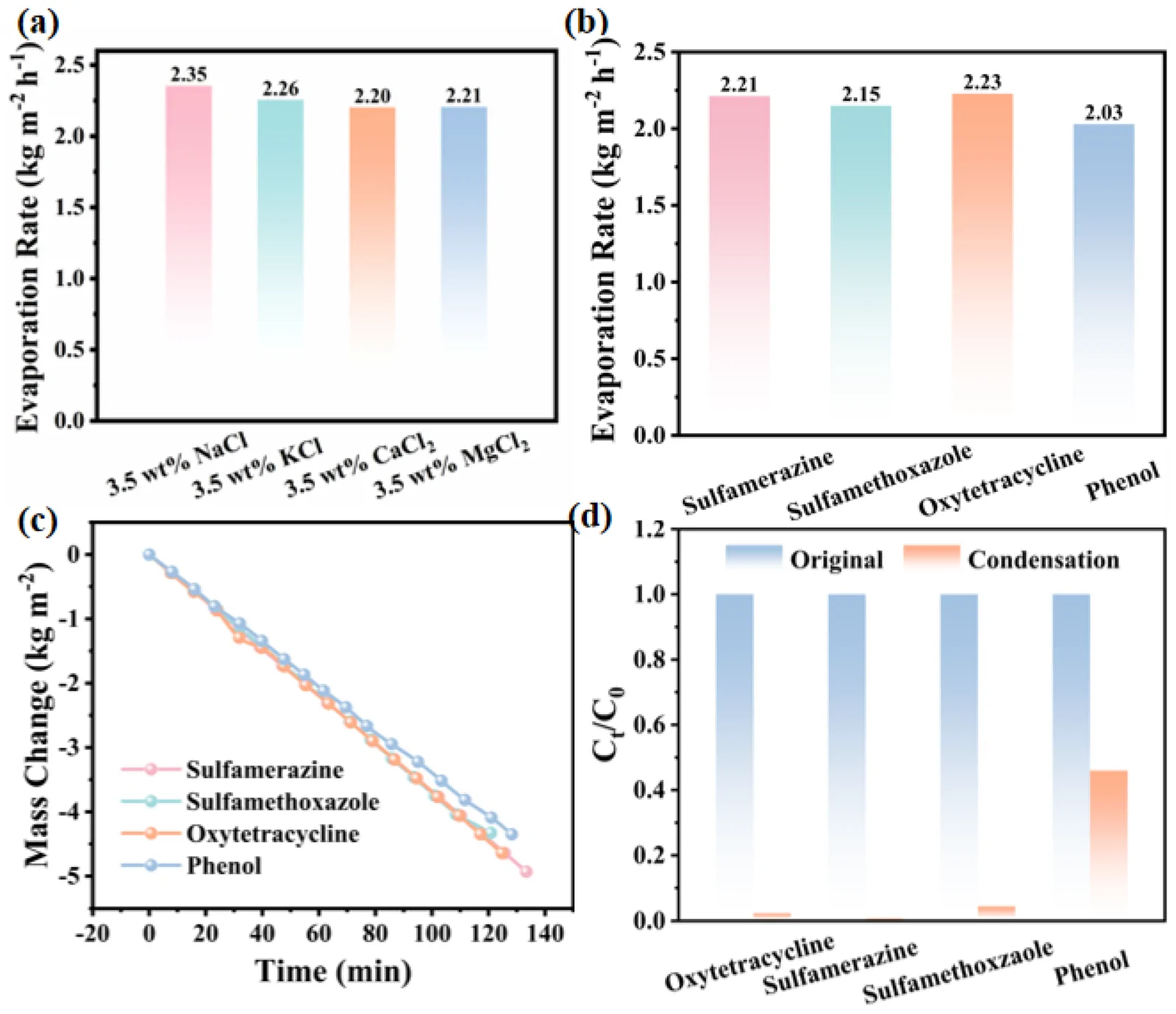
(**a**) Evaporation rate in different salt solutions (NaCl, KCl, MgCl_2_, and CaCl_2_). (**b**) Evaporation rate and (**c**) mass change in CS_2.5_ evaporator in contaminant solutions containing sulfamethoxazole, sulfamethazine, oxytetracycline, and phenol. (**d**) Removal efficiency of the CS_2.5_ evaporator for sulfamethoxazole, sulfamethazine, oxytetracycline, and phenol in water.

The stability and performance of the CS_2.5_ evaporator were further investigated in various salt ions and organic pollutants. To investigate the effect of different salt ion types on the evaporation performance, representative inorganic salts including KCl, MgCl_2_, and CaCl_2_ were selected to construct different ionic systems. The results (Figure 7a) showed that the evaporation rate of the CS_2.5_ evaporator only fluctuated within a small range under various salt solution conditions without significant performance degradation, indicating that the CS_2.5_ evaporator had good compatibility and adaptability to various common ionic systems.

The applicability of the CS_2.5_ evaporator in organic-contaminated water was evaluated using representative organic pollutants, including sulfamethoxazole, sulfamerazine, oxytetracycline, and phenol (Figure 7b,c). The evaporation rate remained relatively stable in the tested organic-contaminant solutions, indicating that these contaminants did not substantially impair evaporation performance over the investigated testing period.

To assess the potential of the CS_2.5_ evaporator for wastewater purification, its removal efficiency for various typical organic pollutants was tested, with a focus on the differential treatment performance between non-volatile and volatile pollutants. The experimental results demonstrated that for non-volatile organic pollutants such as sulfamethoxazole, sulfamerazine, and oxytetracycline, the evaporator exhibited excellent purification capacity, with extremely low pollutant concentrations detected in the condensed water and pollutant removal rates generally exceeding 95% (Figure 7d). This result indicates that the interfacial evaporation system based on the three-dimensional porous matrix can effectively hinder the migration of non-volatile pollutants, ensuring the water quality of the produced water. The phenol rejection efficiency remained below 60% (Figure 7d), consistent with its tendency to partition into the vapor phase during evaporation. This vapor–liquid partitioning allows phenol to migrate with water vapor and subsequently enter the collected condensate. The lower rejection of phenol compared with the tested low-volatility antibiotics highlights the influence of contaminant volatility on separation performance. For feeds containing volatile organic contaminants, an additional purification step could help improve condensate quality. For nonvolatile contaminants, water recovery is accompanied by pollutant enrichment in the residual feed solution. These contaminants are predominantly retained in the feed-side liquid, with possible additional adsorption onto the chitosan framework or accumulation within the lamellar channels. Such retention could alter channel accessibility and water transport during prolonged operation. The stable evaporation rates in Figure 7 indicate maintained evaporation performance under the tested conditions. Further investigation of cumulative pollutant loading and its effects on evaporation would help establish appropriate operating durations and cleaning intervals.

In the present batch configuration, the residual concentrate remains in the feed reservoir and requires separate collection after operation. Future process integration should include the collection of both the concentrate and any pollutant-containing cleaning effluent, followed by treatment selected according to their composition, salinity, and biodegradability. Potential options include adsorption with subsequent management of the spent adsorbent, oxidative treatment, or transfer to a suitable waste-treatment facility. These downstream processes require further evaluation to establish an integrated treatment scheme that combines water recovery with residual-pollutant management.

### 3.5. The Outdoor Evaporation Experiments

**Figure 8:**
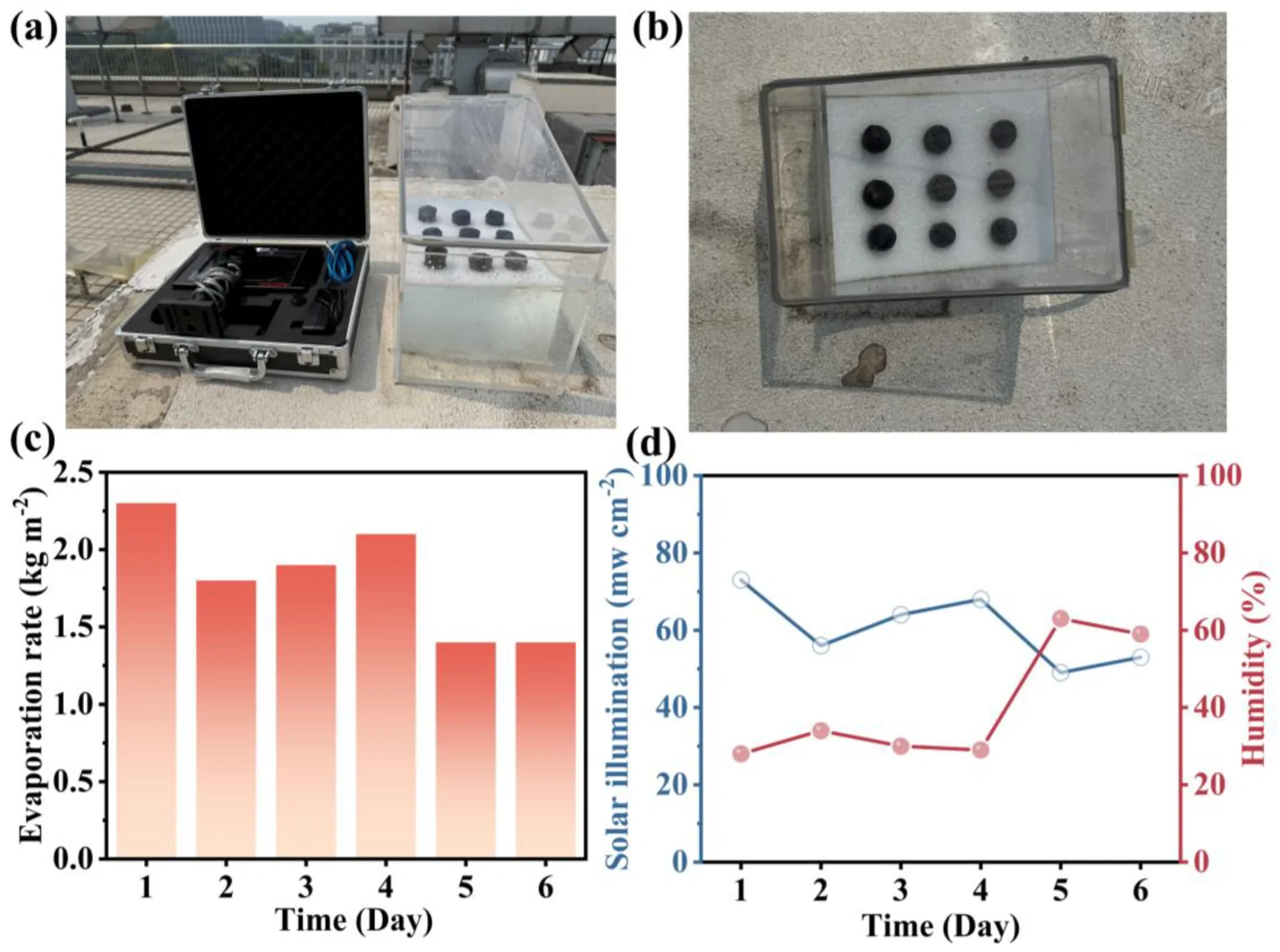
(**a**) Schematic illustration of the outdoor solar water collection system. (**b**) Photograph of the outdoor evaporation device operated on an open rooftop. (**c**) Daily evaporation amount of the CS_2.5_ evaporator under outdoor environmental conditions. (**d**) Variation in solar illumination intensity and relative humidity during six consecutive days of outdoor operation.

Outdoor solar evaporation experiments were further performed to evaluate the practical feasibility of the CS_2.5_ evaporator under real environmental conditions. As shown in Figure 8a,b, the outdoor evaporation system was placed on an open rooftop and directly exposed to natural sunlight. The CS_2.5_ evaporator was supported by a floating polystyrene foam substrate and maintained at the air–water interface, while the transparent inclined cover enabled the generated vapor to condense on the inner surface and flow toward the collection outlet under gravity. Compared with laboratory tests under simulated solar irradiation, the outdoor experiment provided a more realistic assessment because the evaporation process was affected by fluctuating solar intensity, ambient humidity, wind, and heat exchange with the surrounding environment.

The outdoor test was conducted continuously for six days (Figure 8c,d). During this period, the solar illumination varied from approximately 49 to 73 mW cm^−2^, while the relative humidity changed from about 28% to 63%, indicating typical variations in natural weather conditions. Under these fluctuating conditions, the CS_2.5_ evaporator still maintained stable evaporation performance, with the daily evaporation amount remaining in the range of approximately 1.38–2.30 kg m^−2^. On days with relatively strong solar irradiation and low humidity, especially Day 1 and Day 4, higher evaporation performance was obtained. In contrast, the evaporation amount decreased on Day 5 and Day 6, which can be attributed to the reduced solar illumination and significantly increased ambient humidity. Higher humidity weakens the vapor diffusion driving force between the evaporation interface and the surrounding air, thereby suppressing water evaporation to some extent. Nevertheless, no obvious structural collapse or performance failure was observed during the outdoor operation. These results demonstrate that the CS_2.5_ evaporator can adapt to real solar irradiation and environmental fluctuations, further confirming its potential for practical solar-driven freshwater production. Practical deployment also requires consideration of the end-of-life management of CNT-containing aerogels. The monolithic configuration facilitates collection of the spent evaporator, providing a basis for controlled handling and treatment. During aging and disposal, degradation of the chitosan framework and mechanical fragmentation may alter CNT retention and promote release into the surrounding environment. End-of-life management should therefore prioritize intact recovery, contained storage, and treatment procedures appropriate for CNT-containing composites and any retained contaminants. Further assessment of CNT release under relevant aging and disposal conditions would inform the selection of treatment routes and support a more complete evaluation of the environmental performance of this evaporation system.

## 4. Conclusions

In summary, chitosan-based photothermal aerogels with tunable lamellar architectures were fabricated by incorporating carbon nanotubes into chitosan precursors of different concentrations, followed by directional freezing and freeze-drying. Increasing the chitosan concentration reduced the interlayer spacing and altered the water state and transport behavior within the aerogels. Among the prepared samples, CS_2.5_ exhibited the highest proportion of intermediate water and the lowest apparent evaporation enthalpy of 1652.1 kJ kg^−1^, indicating a favorable balance between water–polymer interactions and water mobility. Under 1 sun irradiation, CS_2.5_ achieved the highest evaporation rate of 2.33 kg m^−2^ h^−1^ while maintaining solar absorptance above 97.8% and rapid water wettability. Idealized numerical simulations further indicated that a moderate interlayer spacing promoted a more uniform velocity distribution and a smoother pressure gradient, thereby facilitating continuous water transport. The optimized evaporator maintained stable performance in NaCl solutions with concentrations of up to 15 wt%, different salt systems, organic-contaminated water, and prolonged cycling tests in 3.5 wt% NaCl solution. It also effectively rejected the tested non-volatile organic contaminants, although its separation performance for volatile phenol remained limited. During six consecutive days of outdoor operation, CS_2.5_ delivered daily evaporation yields of 1.38–2.30 kg m^−2^ under fluctuating solar irradiance and relative humidity. These results demonstrate the importance of coordinating lamellar architecture, water-state regulation, and fluid transport and provide a structural design strategy for efficient and environmentally adaptable solar evaporators.

## Data Availability

The original contributions presented in this study are included in the article. Further inquiries can be directed to the corresponding author.

## Supplementary Materials

The following supporting information can be downloaded at: https://www.mdpi.com/article/10.3390/nano16211413/s1, Figure S1: Schematic illustration of the working mechanism and potential applications of the CNT/chitosan lamellar aerogel for solar-driven interfacial evaporation; Figure S2: High-magnification SEM images of the CS2.5 CNT/chitosan composite aerogel at different magnifications: (a) lower-magnification image showing elongated fiber-like structures and dispersed particulate features; (b) higher-magnification image showing the surface morphology and local distribution of fiber-like structures and particle clusters; Figure S3: FTIR spectrum of the CNT/chitosan composite aerogel (CS2.5); Figure S4: Comparison of the apparent evaporation enthalpies of water in the CSx evaporators and bulk water; Figure S5: Surface temperatures of the CSx evaporators under wet and dry conditions: (a) CS1.5, (b) CS2.0, (c) CS2.5, (d) CS3.0, and (e) CS3.5; Figure S6: Digital photographs of the CS2.5 evaporator during continuous evaporation in saline water at (a) 0, (b) 24, (c) 48, and (d) 72 h; Figure S7: Cross-sectional SEM images of CS2.5 after evaporation in 3.5 wt% NaCl solution and subsequent freeze-drying without washing; Figure S8: COMSOL simulation results showing the pore structures of chitosan aerogels with different interlayer spacings: (a) CS2.5 and (b) CS3.5; Figure S9: Mesh quality distribution histogram of the three-dimensional channel model of the chitosan aerogel; Table S1: Performance comparison of aerogel-based photothermal materials for solar interfacial evaporation.
