# Supplementary material for: Interlayer Architecture Modulates Water State and Transport in Chitosan Aerogels for Solar-Driven Interfacial Evaporation
Source: Nanomaterials (Basel). 2026 Nov 3;16(21):1413. doi: 10.3390/nano16211413 (PMC13765083; doi:10.3390/nano16211413)
Supplement: Supplementary file 1 [file nanomaterials-16-01413-s001.zip › nanomaterials-4599295-supplementary.pdf]

Supplemental Information

For

Interlayer Architecture Modulates Water State and Transport in Chitosan Aerogels for Solar-Driven Interfacial Evaporation

Fengxia An[a,b], Zihan Chen[c], Binbin Wang[c], Ke Zhuang[a], Jiayun He[c], Shunde Zou[c], Xiaohuan Meng[a], Enmin Zong[d], Haopeng Luo[c]*, Huan Chen[c]*, Fang Jiang[c]

[a] State Key Laboratory of Low-carbon Smart Coal-fired Power Generation and Ultra-clean Emission, China Energy Science and Technology Research Institute Co. Ltd., Nanjing 210023, China;

[b] Key Laboratory of Energy Thermal Conversion and Control of Ministry of Education, School of Energy and Environment, Southeast University, Nanjing 210096, China;

[c] Key Laboratory of Jiangsu Province for Chemical Pollution Control and Resources Reuse, School of Environmental and Biological Engineering, Nanjing University of Science and Technology, Nanjing 210094, China;

[d] Zhejiang Key Laboratory for Restoration of Damaged Coastal Ecosystems, School of Life Sciences, Taizhou University, Zhejiang Taizhou 318000, China

* Corresponding author: E-mail: luohaopeng@njust.edu.cn, hchen404@njust.edu.cn.

Section S1 Experiments and methods

Section S1.1 Temperature test

Temperature measurement is a critical step in evaluating the photothermal conversion performance of the solar-driven interfacial evaporation device. Two key temperature parameters were monitored using thermocouples during the experiments: the surface temperature of the photothermal aerogel and the temperature of the bulk water. The surface temperature of the aerogel reflected the actual temperature of the material surface under solar irradiation, which could directly characterize the photothermal conversion efficiency of the sample. The bulk water temperature was used to evaluate the degree of heat loss from the evaporation interface to the surrounding water body.

Section S1.2 The design of the water collection device

To verify the purification effect of solar-driven evaporation on high-salinity water, a solar water collection device was designed and fabricated. The material of the transparent cover determined the sunlight transmittance and thereby affected the evaporation rate. After comprehensively considering factors such as cost, weight and light transmittance, quartz glass was ultimately selected as the material for the transparent cover. In addition, the cover of the water collection device was designed with a certain inclination angle, so that the condensed water adhering to the side walls or the cover surface could flow to the bottom of the collection device under gravity and then be smoothly discharged through the outlet.

Section S1.3 Water evaporation test

A xenon lamp was used as the simulated sunlight source, and the irradiation intensity was calibrated using an optical power meter. The beaker containing the water and evaporator was placed on an electronic balance connected to a computer to continuously record the mass change during evaporation. Thermocouples were used to monitor the temperatures at the aerogel surface, within the aerogel, and in the bulk water. The evaporation rate was calculated as:

$$v=\frac{m}{\Delta T\,A} \tag{S1}$$

Where v represents the evaporation rate of water, with the unit of kg m^{-2} h^{-1}; m represents the mass change of water evaporation, with the unit of g; ΔT represents time; and A is the surface area of the photothermal aerogel (cm^{-2}).

Section S1.4 Measurement of enthalpy change of evaporation

The measurement of evaporation enthalpy was carried out at room temperature using the dark-field evaporation method. The detailed process was as follows: the solar-driven interfacial evaporator and water were placed in a sealed container, and the evaporation amounts of the evaporator system and the pure water system were measured separately. Since the pure water and the evaporator were in the same environment, the total energy input received by both systems was identical. Thus, the evaporation enthalpy of water in the evaporator could be calculated using Equation S2:

$$v_{water}\Delta H_{water}=v_{eva}\Delta H_{eva} \tag{S2}$$

Where v_{water} and v_{eva} represent the evaporation rate of pure water and the evaporator respectively, with the unit being kg m-2 h-1. ΔH_{water} and ΔH_{eva} represent the enthalpy change of water evaporation in pure water and the evaporator respectively, with the unit of J g-1. Since ΔH_{water}=2350 J g-1 at 26 °C, it is sufficient to measure the evaporation rates of pure water and the evaporator at 26 °C to calculate the enthalpy change ΔH_{eva} of water in the evaporator.

Section S1.5 COMSOL Multiphysics simulation

To elucidate the microscopic mechanism by which interlayer spacing modulates water transport in layered porous media, three-dimensional pore-scale models of chitosan aerogels with varied interlayer spacings were constructed in COMSOL Multiphysics 6.3, and numerical simulations of aqueous transport were carried out. By employing the porous media flow module, pore connectivity and flow characteristics were analyzed in relation to interlayer spacing. Velocity and pressure fields were quantified to establish correlations between interlayer microstructure, water transport rate, and flow uniformity. The detailed geometric parameters and boundary conditions

applied in these simulations are provided in the Supplementary Information (Figures S8 and S9).

Section S1.6 Analytical Method for Pollutant Concentrations

The concentrations of the target analytes, including sulfamethoxazole, sulfamerazine, oxytetracycline and phenol, were quantified by measuring their absorbance at characteristic wavelengths (typically the maximum absorption wavelength). Absorbance values were converted to corresponding concentrations using pre-established standard curves. Briefly, a series of standard solutions with known concentrations were prepared to derive a linear regression equation between concentration and absorbance. The absorbance of pretreated samples was then measured under identical conditions, and the pollutant concentration was determined by interpolation against the standard curve, from which the removal rate or degradation efficiency was further calculated.

Section S1.7 COMSOL Simulation Modeling

1. Reconstruction of 3D Pore Structure

Fig. S8presents the reconstructed 3D pore structures of chitosan aerogels at different chitosan concentrations. The $CS_{2.5}$ aerogel exhibits a continuous, uniform, and highly interconnected layered pore network, which provides an ideal structural foundation for efficient water transport. In contrast, as the chitosan concentration increases to $CS_{3.5}$, the interlayer spacing is excessively compressed, resulting in narrowed channels and local blockages, which fundamentally hinder the smooth flow of water.

2. Mesh Quality Evaluation

The accuracy and reliability of COMSOL simulations are guaranteed by rigorous mesh generation and quality evaluation. As shown in Fig. S9 the model is discretized with refined free tetrahedral meshes, and the vast majority of mesh elements are concentrated in the high-quality region (>0.7), with an extremely low proportion of low-quality distorted elements. The excellent overall mesh quality, free of severe distortion or slender elements, ensures the convergence and reliability of subsequent

flow field and pressure field simulations, laying a high-precision model foundation for revealing the water transport mechanism in aerogel pores.

During the simulation, peristaltic flow was used to simulate microscopic flow under different pore conditions, and its diffusion rate within the pores was calculated according to the following equation:

$$\rho(\mathbf{u}\cdot\nabla)\mathbf{u}=\nabla\cdot[-p\mathbf{I}+\boldsymbol{\tau}]+\mathbf{F} \tag{S3}$$

$$\nabla\cdot\mathbf{u}=0 \tag{S4}$$

$$\boldsymbol{\tau}=\mu(\nabla\mathbf{u}+(\nabla\mathbf{u})^{T}) \tag{S5}$$

where ρ represents the fluid density (kg m^{-3}), u is the fluid velocity vector (m s^{-1}), p denotes the fluid pressure (Pa), I is the second-order identity tensor, τ represents the viscous stress tensor (Pa), μ is the dynamic viscosity of water (Pa s), and F denotes the external body force per unit volume (N m^{-3}). The superscript T represents the transpose operator.

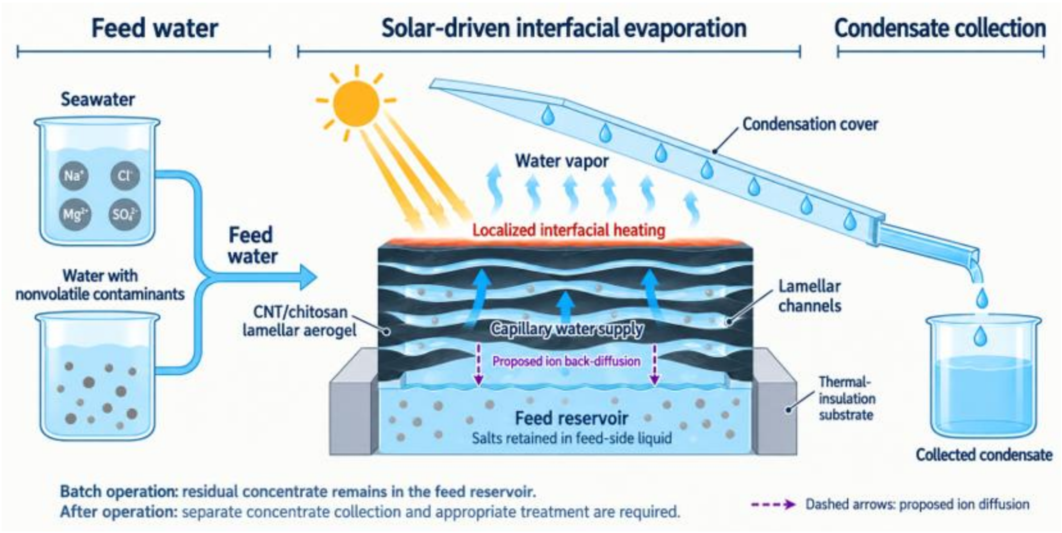

Figure S1 Schematic illustration of the working mechanism and potential applications of the CNT/chitosan lamellar aerogel for solar-driven interfacial evaporation.

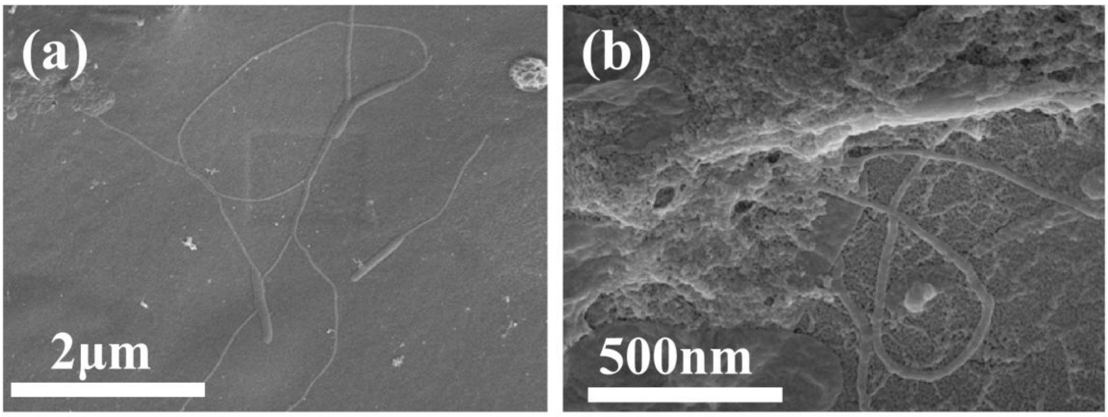

Figure S2. High-magnification SEM images of the $CS_{2.5}$ CNT/chitosan composite aerogel at different magnifications: (a) lower-magnification image showing elongated fiber-like structures and dispersed particulate features; (b) higher-magnification image showing the surface morphology and local distribution of fiber-like structures and particle clusters.

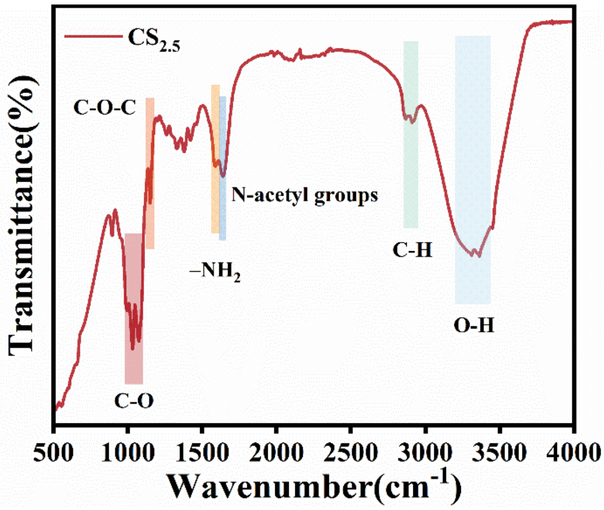

Figure S3. FTIR spectrum of the CNT/chitosan composite aerogel ($CS_{2.5}$).

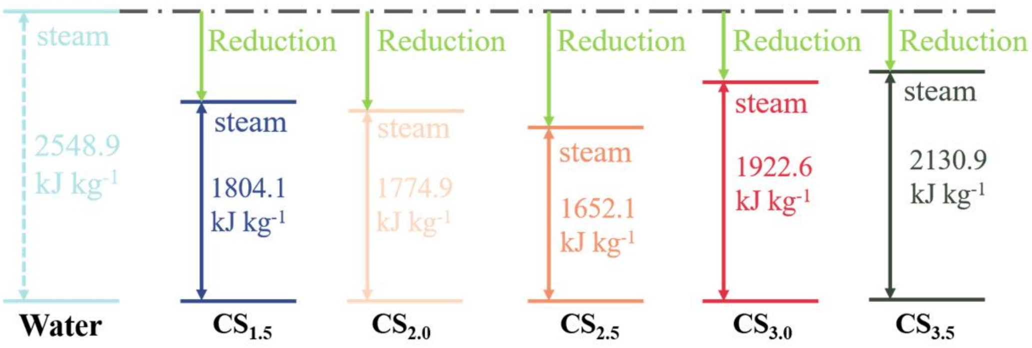

Figure. S4 Comparison of the apparent evaporation enthalpies of water in the CS_x evaporators and bulk water.

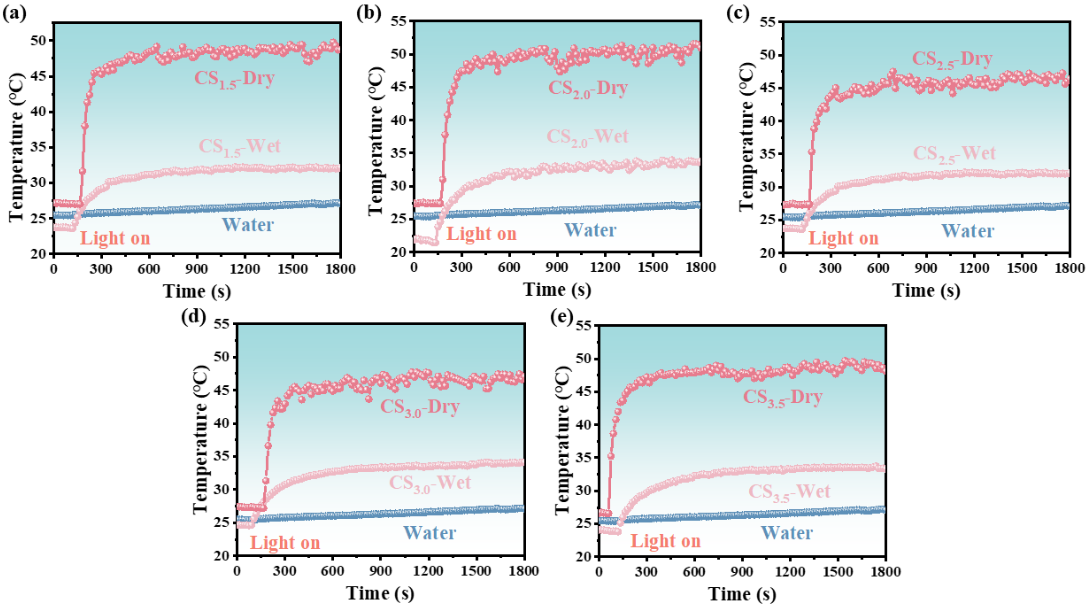

Figure. S5 Surface temperatures of the CS_x evaporators under wet and dry conditions: (a) $CS_{1.5}$, (b) $CS_{2.0}$, (c) $CS_{2.5}$, (d) $CS_{3.0}$, (e) $CS_{3.5}$.

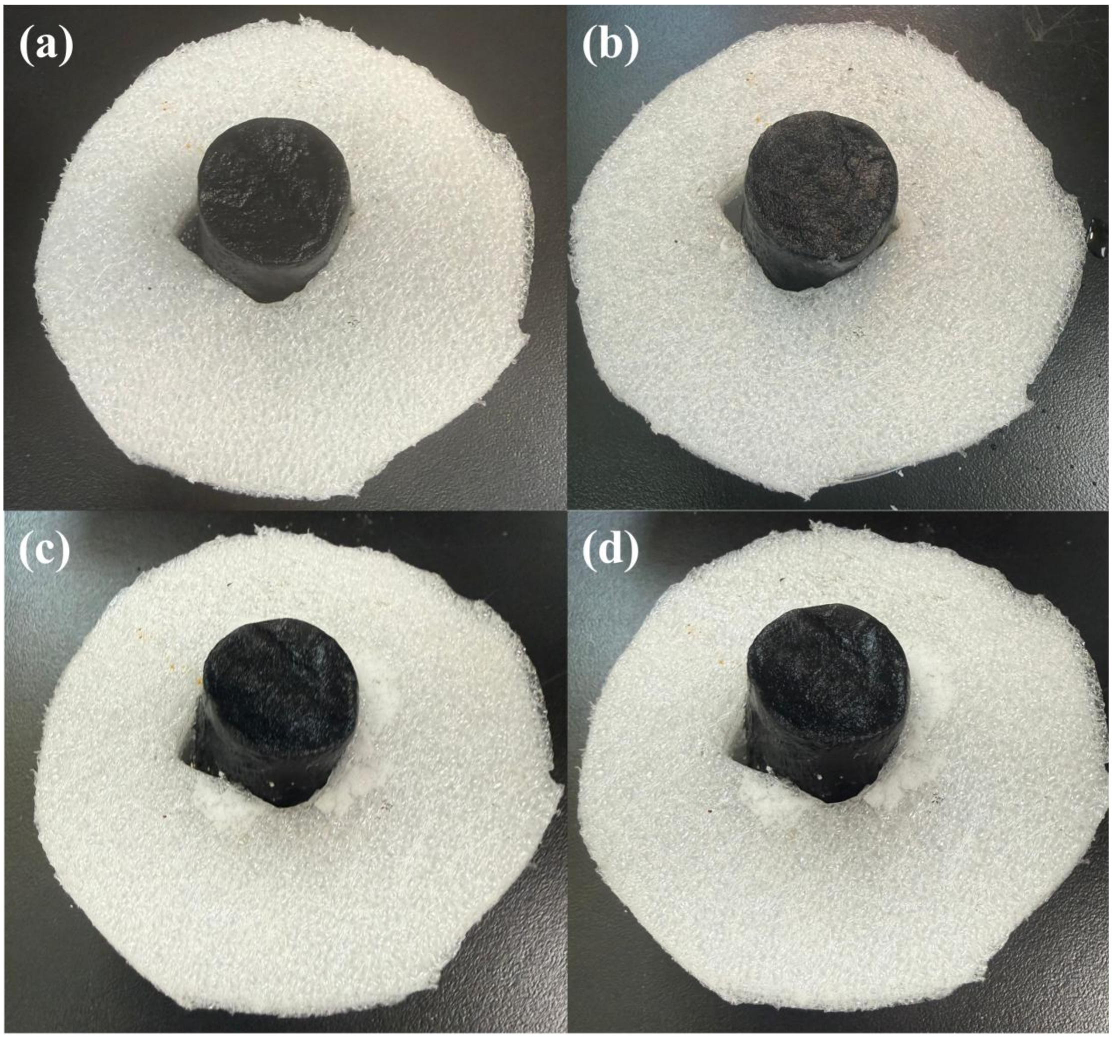

Figure S6. Digital photographs of the $CS_{2.5}$ evaporator during continuous evaporation in saline water at (a) 0, (b) 24, (c) 48, and (d) 72 h.

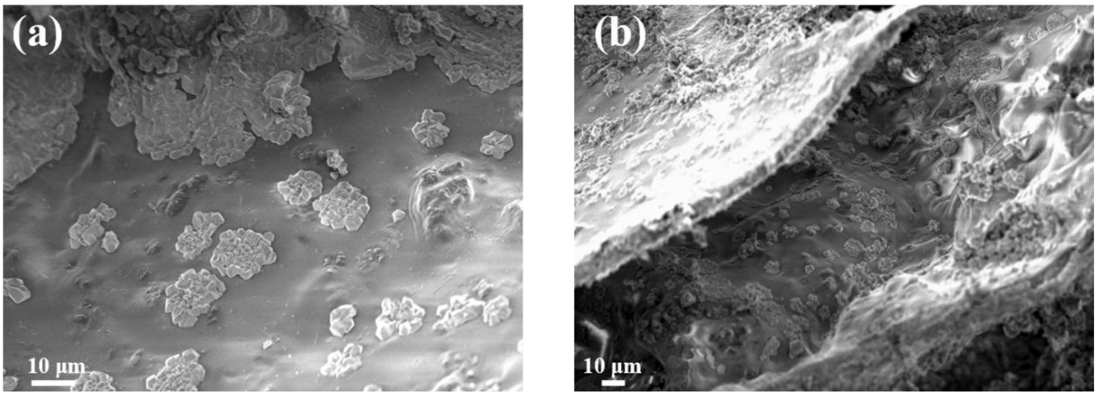

Figure S7. Cross-sectional SEM images of $CS_{2.5}$ after evaporation in 3.5 wt% NaCl solution and subsequent freeze-drying without washing.

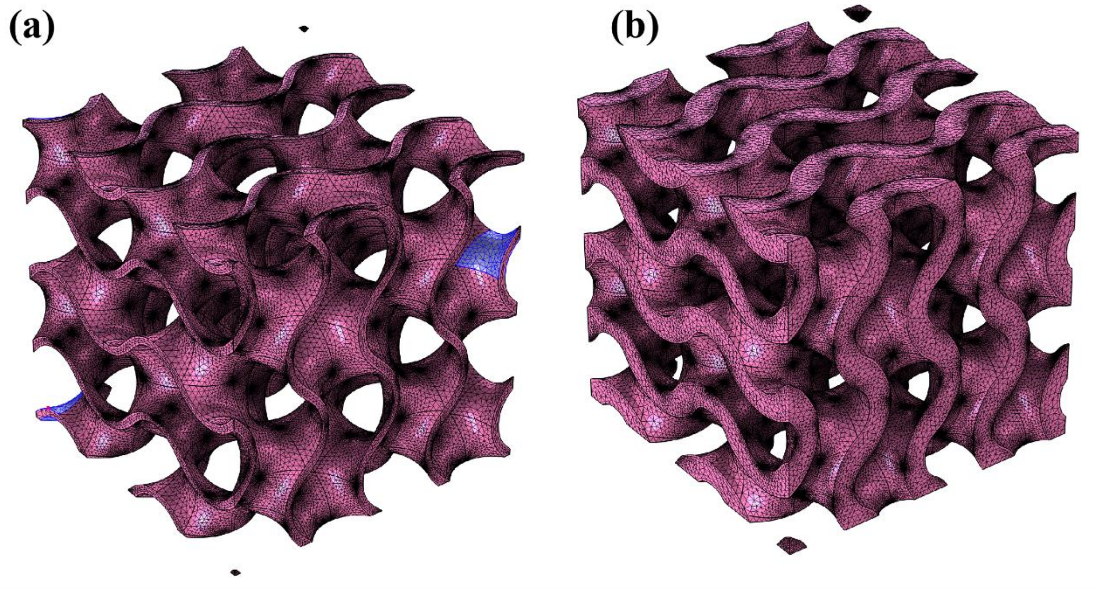

Figure. S8 (a) $CS_{2.5}$ and (b) $CS_{3.5}$ show the COMSOL simulation results of the pore structure of chitosan aerogels with different interlayer spacings.

(a)

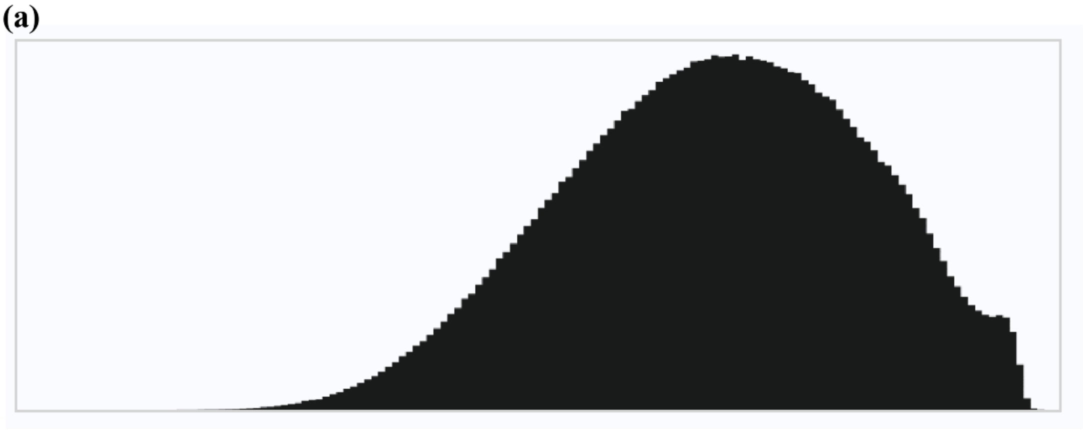

Figure. S9 Mesh quality distribution histogram of the 3D channel model of chitosan aerogel.

Table S1 Performance comparison of aerogel-based photothermal materials for solar interfacial evaporation.

Material	Evaporation Rate ($kg\ m^{-2}\ h^{-1}$)	Light Absorption / Efficiency	Key Features	Ref.
A-$Ti_3C_2T_x$/CNT hybrid aerogel	2.10	95.68% / 93.4%	Anisotropic, vertically aligned channels	[36]
FT-CNT-CMC composite aerogel	1.94	96–98% / 92.8%	Bilayer structure (hydrophilic/hydrophobic)	[37]
Cellulose/chitosan composite aerogel	3.29 (pure water) / 3.57 (3.5 wt% brine)	— / 86.2%	All-biobased, low evaporation enthalpy	[35]
Lignocellulose/MXene composite aerogel	2.86	— / 93.36%	Biomass-based, pore size optimization	[38]
mMWCNTs/CS-MXene/XSBR aerogel/film composite	2.44	>96.2% / 92.2%	Double-layer asymmetric structure	[39]
Chitosan aerogel-carbon nanotubes (CA-CNT)	1.55	95.04% / 97.15%	Double-layer structure, vertical pores	[40]
Lignin-mediated chitosan/lignin hybrid aerogel	3.569	— / 92.05%	Lignin-regulated pore size, Ag NPs plasmon-enhanced	[41]
$CS_{2.5}$ evaporator (This work)	2.33	97.81%	Chitosan/MWCNTs, interlayer spacing optimization	This work
